# Peroneal neuropathy caused by an extraneural ganglion cyst in the supracondylar area of the femur: A case report

**DOI:** 10.1097/MD.0000000000022123

**Published:** 2020-09-11

**Authors:** Jaehoon Sim, Hyunseok Kwak, Soonchul Lee, Kyunghoon Min

**Affiliations:** a Department of Rehabilitation Medicine, CHA Bundang Medical Center, CHA University School of Medicine, Seongnam, Korea; b Rehabilitation and Regeneration Research Center, School of Medicine, Seongnam, Korea; c Department of Orthopaedic Surgery, CHA Bundang Medical Center, CHA University School of Medicine, Seongnam, Korea.

**Keywords:** peroneal neuropathy, ganglion cyst, extraneural, distal thigh

## Abstract

**Rationale:**

Peroneal neuropathy is the most common type of peripheral neuropathy in the lower extremities. The peroneal nerve is usually compressed at the lateral aspect of the fibular head. Compression by ganglion cysts are one of the numerous underlying etiologies for peroneal nerve neuropathy and are most frequently located around the fibular neck and proximal tibiofibular joint. To the best of our knowledge, this is the first report of an extraneural ganglion cyst located at the level of the distal thigh that resulted in compressive peroneal neuropathy.

**Patient concerns:**

We report a case of a 56-year-old man with sudden onset of left foot drop and gait disturbance caused by an extraneural ganglion cyst located in the popliteal fossa.

**Diagnosis:**

Electrodiagnosis (EDX) suggested a peroneal nerve lesion. Subsequently, diagnostic ultrasonography (USG) revealed a cystic mass located within the left side of the supracondylar area of femur. Further magnetic resonance imaging confirmed that the mass was located at the proximal of popliteal fossa.

**Interventions:**

Surgical excision was performed using a direct posterior approach. The cystic mass was compressing the common peroneal nerve, and was carefully and completely removed ensuring that all nerve branches were protected.

**Outcomes:**

A histopathologic evaluation confirmed the diagnosis of a ganglion cyst. There were no postoperative complications. Two months after the surgery, follow-up USG revealed no evidence of cyst recurrence or residual lesions. Six months after the surgery, the ankle dorsiflexor motor power improved and he experienced less pain and hypoesthesia.

**Lessons:**

Physicians should bear in mind that the peroneal neuropathy can occur because of the ganglion cyst in the distal thigh. The thorough evaluation of EDX and USG is crucial for the early diagnosis and surgical intervention, although there is no abnormal finding around the fibular neck.

## 1 Introduction

Foot drop can be ascribed to peripheral lesions in the L5 nerve root, sciatic, or peroneal nerves.^[1]^ Peroneal neuropathy is the most common etiology of foot drop, and it must be distinguished from L5 radiculopathy.^[2]^ Globally, the lifetime prevalence of lumbar radiculopathies ranges from 1.2% to 43%, with L5 being the most frequently affected nerve root.^[3,4]^ Moreover, L5 radiculopathy-induced foot drop may indicate the presence of a progressive neurological deficit, which usually requires surgical evaluation.^[5]^

Peroneal neuropathy has numerous underlying etiologies and is commonly caused by compression at the level of fibular neck, which can result from prolonged immobilization during deep sleep and anesthesia, in addition to trauma and stretch injury.^[6]^ However, in rare cases, it can also be caused by compressive mass lesion around the knee joint, such as cyst or nerve sheath tumors.^[6,7]^

Cystic lesions around the knee joint can be categorized into ganglion, synovial, and meniscal cysts.^[8]^ Ganglion cysts are benign tumors that usually develop in underlying joint capsules or tendon sheaths and peripheral nerves, and characteristically contain mucinous fluid. They differ from other types of cyst in that they are not lined by synovial cells and are not associated with meniscal tears.^[8]^

Since the first case report by Sultan in 1921 of a compression peroneal neuropathy caused by an intraneural ganglion cyst, limited reports have described similar cases.^[9–15]^ Across previous reports, the most frequent location for cystic mass development was around the fibular neck and proximal tibiofibular joint. Moreover, a few studies have reported the development of peroneal neuropathy caused by intraneural-type cysts formed in the popliteal fossa.^[16,17]^ To the best of our knowledge, no reports have described a case of common peroneal neuropathy caused by an extraneural ganglion cyst located at the proximal site of popliteal fossa.

In this report, we describe a patient with common peroneal nerve palsy who presented with foot drop caused by compression by an extraneural ganglion cyst at the supracondylar area of femur.

## 2 Case report

**Figure 1:**
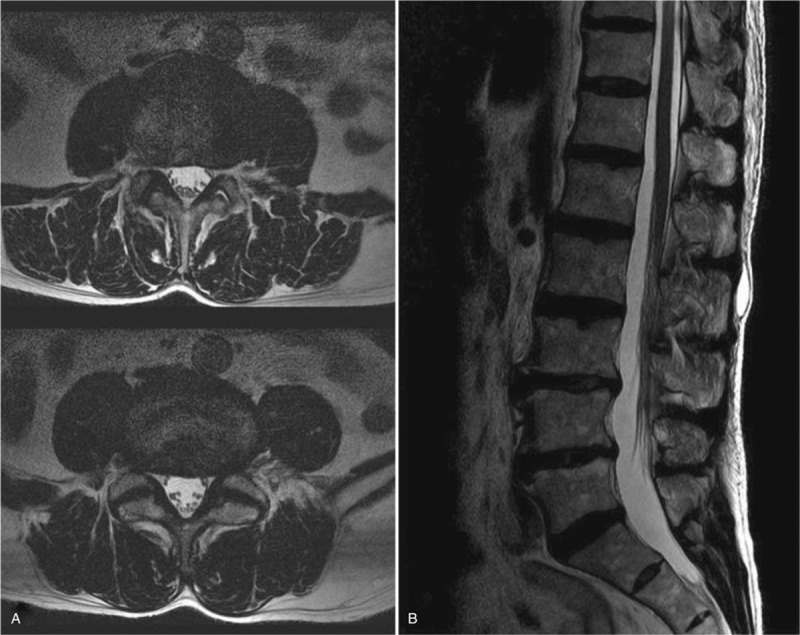
Axial (A) and sagittal (B) views of T2-weighted magnetic resonance images of the lumbar spine show mild disc protrusions of L3-4 and L4-5.

A 56-year-old male welder presented to a hospital with sudden onset of left foot drop. He underwent lumbar magnetic resonance imaging (MRI) and mild disc protrusions of L3-4 and L4-5 were observed (Fig. 1). The initial diagnosis was L5 radiculopathy with profound ankle dorsiflexor weakness. Spine operation was planned and electrodiagnosis (EDX) was conducted to determine the exact location of lesion and to differentiate peripheral nerve lesions before surgery in another clinic (Table 1).

**Table 1:**
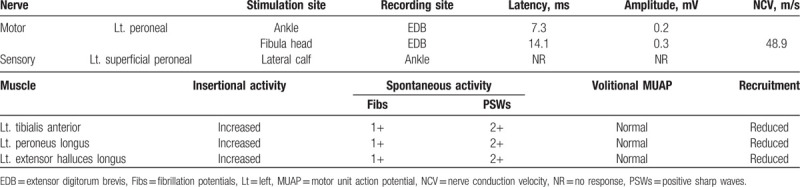
Electrodiagnostic analysis 9 days after the onset of symptoms.

The patient had been experiencing foot drop for 9 days and initial EDX results revealed abnormal spontaneous activity (ASA) in the left tibialis anterior (TA), peroneus longus (PL), and extensor hallucis longus (EHL). These findings suggested that the patient's foot drop was more likely due to peroneal nerve injury than lumbar radiculopathy. ASA, such as fibrillation, usually appears within 5 to 6 weeks after the onset of the lesion in radiculopathy.^[18]^ Consequently, the surgery was cancelled.

The next day, the patient was referred to our outpatient clinic for peroneal neuropathy. He complained of numbness in the area below the knee and especially in the dorsal aspect of his foot. These symptoms began 10 days prior and he did not recall any history of trauma. On physical examination, muscle manual testing (MMT) of the TA and EHL revealed a trace grade, while the peroneus muscle showed poor plus grade. There were no signs of TA or peroneal muscle atrophy. Soft tissue swelling was not palpable around the fibular head and popliteal fossa. The palpation of the lateral aspect of the knee showed no degree of tenderness. The Tinel sign over the fibular head was negative. The patient's ankle and knee jerks were normal.

EDX, performed on the day of the patient's visit to our outpatient clinic, showed common peroneal neuropathy around the left knee with significant axonal denervation in the TA and PL. Compared with the contralateral limb, the compound muscle action potential (CMAP) amplitude was reduced by greater than 70% in the left lower limb distal muscles, which are innervated by the common peroneal nerve. However, because a conduction block across fibular head was not observed, we considered that the lesion was proximal to the stimulation site. Consequently, a diagnostic ultrasonography (USG) was performed to identify the lesion, which was compressing the peroneal nerve. However, we did not find any lesion around the fibular neck and proximal tibiofibular joint, where compression of peroneal nerve is most commonly found.

**Figure 2:**
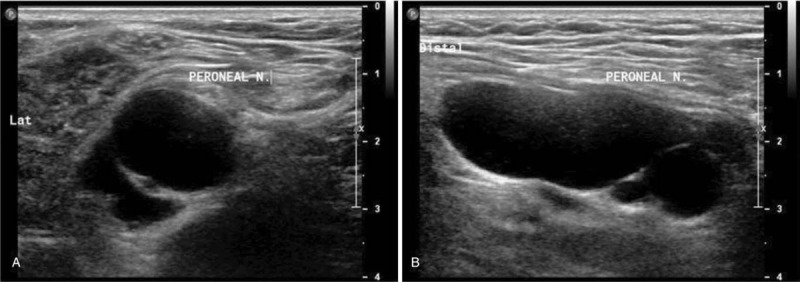
Transverse (A) and longitudinal (B) views of ultrasonography show an approximately 19 × 16 × 40 mm sized hypoechoic cystic mass located at the upper lateral component of the left popliteal fossa.

**Figure 3:**
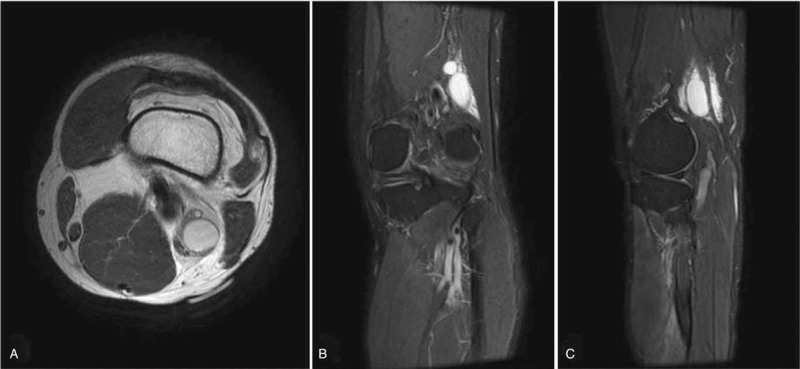
Axial (A), coronal (B), and sagittal (C) views of T2-weighted magnetic resonance images show an approximately 18 × 17 × 41 mm sized multilobulated cystic lesion, which was located anteromedial to the common peroneal nerve in the supracondylar area of the left femur.

At the 1-week follow-up, the patient's symptoms did not improve. As compression of the peroneal nerve can occur anywhere along its axonal tract, we investigated whether the compression site was located proximally to the knee. A follow-up USG revealed an approximately 19 × 16 × 40 mm sized cystic mass located in the upper lateral component of the popliteal fossa (Fig. 2). Surgical excision was planned and MRI was evaluated to find the anatomical relation between mass and peroneal nerve. The images revealed the presence of an approximately 18 × 17 × 41 mm sized multilobulated cystic lesion, which was located anteromedial to the common peroneal nerve and between the common peroneal and tibial nerves on the left side of the supracondylar area of the femur (Fig. 3).

**Figure 4:**
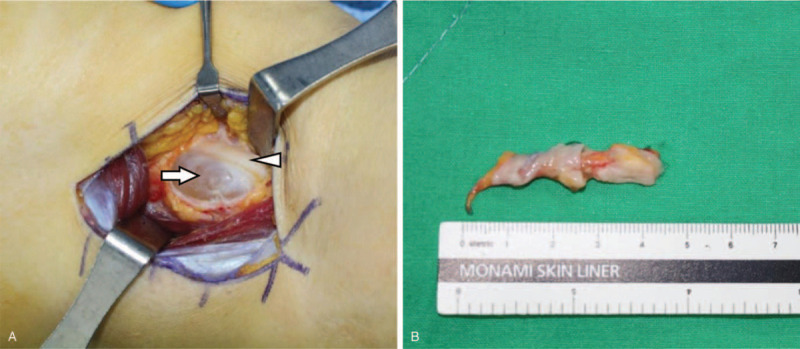
(A) An intraoperative photograph from the supracondylar area of femur showing the cystic mass that was compressing the common peroneal nerve (Arrow head: Peroneal nerve, Arrow: Ganglion cyst). (B) The ganglion cyst was completely excised from common peroneal nerve compression site.

Surgical excision was performed using a direct posterior approach of popliteal fossa using curvelinear incision under general anesthesia. We found the cystic mass after traction of hamstring muscle. The cystic mass was compressing the common peroneal nerve from the medial side of the supracondylar region on the femur resulting the swelling of the nerve. The mass was carefully and completely removed ensuring that all nerve branches were protected with neurolysis (Fig. 4). The mass was not connected to the knee joint. A histopathologic evaluation confirmed the diagnosis of a ganglion cyst. There were no postoperative complications.

**Figure 5:**
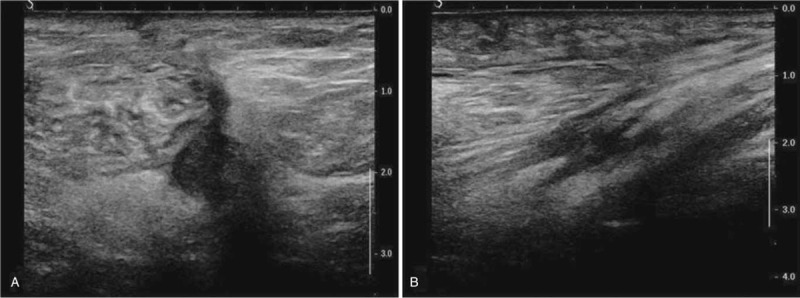
Two months after surgery, follow-up transverse (A) and longitudinal (B) ultrasonography images show no evidence of recurrent or residual lesions, and only show typical postoperative changes in the posterolateral aspect of the left knee.

Two months after the surgery, follow-up USG revealed no evidence of cyst recurrence or residual lesions (Fig. 5). Moreover, the amplitude of left peroneal nerve CMAP improved mildly on follow-up EDX. Six months after surgery, the patient's pain and hypoesthesia resolved. MMT of the TA and EHL improved to a fair grade.

## 3 Discussion

According to Malghem et al,^[8]^ knee cysts can be classified into ganglion, synovial, and meniscal cysts (Table 2). Table 2 summarizes case reports of the cyst around knee that were previously reported according to the classification.^[10,11,14,16,19–23]^ To our knowledge, our case is the first report of compressive peroneal neuropathy caused by extraneural type of ganglion cyst in the supracondylar area of femur. Ganglion cyst compressions of the peroneal nerve can be classified as intraneural or extraneural lesions.^[24]^ Most ganglion cysts, which cause peroneal nerve compression, are of an intraneural type.^[25]^ This type of cyst is often related to a history of traumatic knee injury.^[26]^ Interestingly, our patient did not sustain any direct traumatic injury to his knee. However, considering that he had worked as a welder for decades and had sustained a hyperflexion of his knee, his extraneural cyst may have resulted from repeated microtraumas.

**Table 2:**
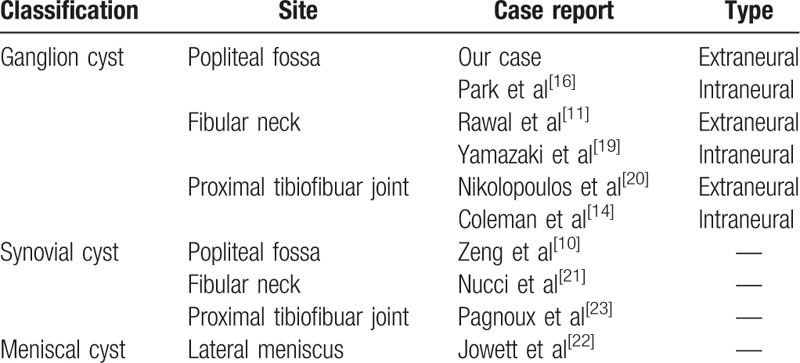
Cystic lesions around the knee classified by Malghem et al^[8]^.

In this case, EDX, USG, and MRI were used for diagnosis and treatment. Standard plain radiographs are of little value to detect of soft tissue lesions such as ganglion cyst despite of being useful in excluding bony abnormalities or fracture of fibular neck.^[11]^ EDX can be helpful for locating the compressive lesion and can provide information regarding the severity of the neuropathy, which can determine the neurological and functional prognosis.^[12]^ In addition, EDX can discriminate other peripheral nerve lesions including plexopathy or radiculopathy.^[27]^ Both USG and MRI can be used as diagnostic methods. USG can be easily performed to rule out space occupying lesions in outpatient clinics due to its portability and cost benefit.^[28]^ It is particularly useful for showing the cystic nature of mass and distinguishing it from solid tumors.^[11]^ MRI can be used to reveal the characteristics of the lesions and to identify the relationship between the compressive lesion with the joint and surrounding anatomic structures.^[12]^

There have been some studies where cyst aspiration with corticosteroid injection was used as a treatment for cyst around the knee,^[23,29]^ but it has been accepted as a less favorable method because of the high recurrence rate and the risk of nerve damage.^[12]^ In many previous studies, surgical excision is a standard treatment for ganglion cyst. In general, it has been recommended to perform early surgical excision in case of symptoms, but Fansa et al^[30]^ recommended surgical intervention to prevent delayed damage even if there are no neurological symptoms. Therefore, even minor symptoms in early phase should be considered as an indication of surgical excision following correct diagnosis.

In conclusion, peroneal neuropathy caused by an extraneural ganglion cyst is a rare and often misleading condition. When determining the etiology of peroneal neuropathy, a ganglion cyst around the knee should also be considered among the differential diagnoses. Using a combination of EDX, USG, and MRI can help in the formulation of an accurate differential diagnosis in difficult-to-diagnose cases. Although the optimal timing of surgical removal has not been described in the literature, early intervention, before axonal damage, is recommended.^[15,17,31]^ Careful examination for diagnosing the ganglion cyst and providing early surgical intervention can lead to a satisfactory recovery.

## Author contributions

**Conceptualization:** Kyunghoon Min.

**Data curation:** Jaehoon Sim, Hyunseok Kwak.

**Supervision:** Soonchul Lee, Kyunghoon Min.

**Writing – original draft:** Jaehoon Sim.

**Writing – review & editing:** Soonchul Lee, Kyunghoon Min.

## References

[1] Stevens F Weerkamp NJ Cals JW Foot drop. BMJ 2015;350:h1736. 25918354 10.1136/bmj.h1736

[2] Stewart JD Foot drop: where, why and what to do? Pract Neurol 2008;8:158 69. 18502948 10.1136/jnnp.2008.149393

[3] Konstantinou K Dunn KM Sciatica: review of epidemiological studies and prevalence estimates. Spine (Phila Pa 1976) 2008;33:2464 72. 18923325 10.1097/BRS.0b013e318183a4a2

[4] Dydyk AM Das MJ Radicular Back Pain. Treasure Island: StatPearls Publishing; 2019.

[5] Tarulli AW Raynor EM Lumbosacral radiculopathy. Neurol Clin 2007;25:387 405. 17445735 10.1016/j.ncl.2007.01.008

[6] Masakado Y Kawakami M Suzuki K Clinical neurophysiology in the diagnosis of peroneal nerve palsy [in Japanese]. Keio J Med 2008;57:84 9. 18677088 10.2302/kjm.57.84

[7] Poage C Roth C Scott B Peroneal nerve palsy: evaluation and management. J Am Acad Orthop Surg 2016;24:1 0. 26700629 10.5435/JAAOS-D-14-00420

[8] Malghem J Vande Berg B Lebon C Ganglion cysts of the knee: articular communication revealed by delayed radiography and CT after arthrography. AJR Am J Roentgenol 1998;170:1579 83. 9609177 10.2214/ajr.170.6.9609177

[9] Sultan C Ganglion der nervenscheide des nervus peroneus. Zentralbl Chir 1921;48:963 5.

[10] Zeng X Xie L Qiu Z Compression neuropathy of common peroneal nerve caused by a popliteal cyst: a case report. Medicine 2018;97:e9922. 29668644 10.1097/MD.0000000000009922 PMC5916662

[11] Rawal A Ratnam KR Yin Q Compression neuropathy of common peroneal nerve caused by an extraneural ganglion: a report of two cases. Microsurgery 2004;24:63 6. 14748028 10.1002/micr.10203

[12] Zumrut M Demirayak M Kucukapan A An unusual cause of foot drop: peroneal extraneural ganglion cyst. Pak J Med Sci 2016;32:1047 50. 27648065 10.12669/pjms.324.9998 PMC5017076

[13] Greer-Bayramoglu RJ Nimigan AS Gan BS Compression neuropathy of the peroneal nerve secondary to a ganglion cyst. Can J Plast Surg 2008;16:181 3. 19721802 10.1177/229255030801600307 PMC2691018

[14] Coleman SH Beredjeklian PK Weiland AJ Intraneural ganglion cyst of the peroneal nerve accompanied by complete foot drop: a case report. Am J Sports Med 2001;29:238 41. 11292053 10.1177/03635465010290022101

[15] Muramatsu K Hashimoto T Tominaga Y Unusual peroneal nerve palsy caused by intraneural ganglion cyst: pathological mechanism and appropriate treatment. Acta Neurochir 2013;155:1757 61. 23702792 10.1007/s00701-013-1768-z

[16] Park SH Do HK Jo GY Compressive peroneal neuropathy by an intraneural ganglion cyst combined with L5 radiculopathy: a case report. Medicine (Baltimore) 2019;98:e17865. 31689879 10.1097/MD.0000000000017865 PMC6946429

[17] Tehli O Celikmez RC Birgili B Pure peroneal intraneural ganglion cyst ascending along the sciatic nerve. Turk Neurosurg 2011;21:254 8. 21534214 10.5137/1019-5149.JTN.2660-09.1

[18] Wilbourn AJ Aminoff MJ AAEM minimonograph 32: the electrodiagnostic examination in patients with radiculopathies. Muscle Nerve 1998;21:1612 31. doi: 10.1002/(sici)1097-4598(199812)21:12<1612::aid-mus2>3.0.co;2-0. 9843062 10.1002/(sici)1097-4598(199812)21:12<1612::aid-mus2>3.0.co;2-0

[19] Yamazaki H Saitoh S Seki H Peroneal nerve palsy caused by intraneural ganglion. Skeletal Radiol 1999;28:52 6. 10068077 10.1007/s002560050473

[20] Nikolopoulos D Safos G Sergides N Deep peroneal nerve palsy caused by an extraneural ganglion cyst: a rare case. Case Rep Orthop 2015;2015:861697. 25632363 10.1155/2015/861697 PMC4302346

[21] Nucci F Artico M Santoro A Intraneural synovial cyst of the peroneal nerve: report of two cases and review of the literature. Neurosurgery 1990;26:339 44. 2155391 10.1097/00006123-199002000-00028

[22] Jowett AJ Johnston JF Gaillard F Lateral meniscal cyst causing common peroneal palsy. Skeletal Radiol 2008;37:351 5. 18193217 10.1007/s00256-007-0430-3

[23] Pagnoux C Lhotellier L Marek JJ Synovial cysts of the proximal tibiofibular joint: three case reports. Joint Bone Spine 2002;69:331 3. 12102284 10.1016/s1297-319x(02)00403-7

[24] Ghossain M Mohasseb G Dagher F Compression of the common peroneal nerve by a synovial cyst [in French]. Neurochirurgie 1987;33:412 4. 3696364

[25] Sobol GL Lipschultz TM Successful surgical treatment of an intraneural ganglion of the common peroneal nerve. Am J Orthop 2015;44:E123 6. 25844595

[26] Yazid Bajuri M Tan BC Das S Compression neuropathy of the common peroneal nerve secondary to a ganglion cyst. La Clin Ter 2011;162:549 52. 22262327

[27] Alsahhaf A Renno W Ganglion cyst at the proximal tibiofibular joint in a patient with painless foot drop. Pain Physician 2016;19:1147 60. 27906945

[28] Peng PW Shankar H Ultrasound-guided interventional procedures in pain medicine: a review of anatomy sonoanatomy, and procedures part V: knee joint. Reg Anesth Pain Med 2014;39:368 80. 25075456 10.1097/AAP.0000000000000135

[29] Jerome D McKendry R Synovial cyst of the proximal tibiofibular joint. J Rheumatol 2000;27:1096 8. 10782844

[30] Fansa H Plogmeier K Gonschorek A Common peroneal nerve palsy caused by a ganglion: case report. Scand J Plast Reconstr Hand Surg 1998;32:425 8. 10.1080/02844319850158525 9862111

[31] Oshima Y Fetto JF Permanent motor function loss by delayed treatment of peroneal intraneural ganglion. Bull Hosp Jt Dis 2016;74:306 8. 27815955

